# Targeted Next-Generation Sequencing in the Molecular Diagnosis of Severe Combined Immunodeficiency

**DOI:** 10.3390/medicina61091644

**Published:** 2025-09-11

**Authors:** Evangelos Bakaros, Styliani Sarrou, Antonios Gkantaras, Alexia Matziri, Achilleas P. Galanopoulos, Konstantina Charisi, Athanasios Bangeas, Anna Taparkou, Eleni Papadimitriou, Varvara A. Mouchtouri, Fani Kalala, Christos Hadjichristodoulou, Matthaios Speletas, Evangelia Farmaki

**Affiliations:** 1 Department of Immunology and Histocompatibility, Faculty of Medicine, University of Thessaly, 41500 Larissa, Greece; ebakaros@uth.gr (E.B.); ssarrou@uth.gr (S.S.); acgalanopoulos@uth.gr (A.P.G.); fkalala@uth.gr (F.K.); 2 Pediatric Immunology and Rheumatology Referral Centre, First Department of Pediatrics, Aristotle University, “Hippokration” General Hospital, 54642 Thessaloniki, Greece; agkantar@auth.gr (A.G.); ktcharisi@auth.gr (K.C.); th.mpagg@gmail.com (A.B.); annatapark@gmail.com (A.T.); epapadimitriu@gmail.com (E.P.); farmakg@auth.gr (E.F.); 3 Laboratory of Hygiene and Epidemiology, Faculty of Medicine, University of Thessaly, 41222 Larissa, Greece; alexmatz@uth.gr (A.M.); mouchtourib@uth.gr (V.A.M.); xhatzi@uth.gr (C.H.)

**Keywords:** NGS, SCID, ADA-SCID, ARTEMIS-SCID, X-SCID

## Abstract

*Background and Objectives*: Severe combined immunodeficiency (SCID) represents a group of rare and potentially fatal monogenic disorders arising from pathogenic variants in a broad spectrum of genes. Diagnostic delays beyond the first few months of life have been associated with poor overall survival and hematopoietic stem cell transplantation (HSCT) outcomes. Therefore, the aim of our study was to apply an NGS assay enabling the rapid and reliable diagnosis of SCID. *Materials and Methods*: We developed a targeted NGS panel of 30 genes implicated in the pathogenesis of most SCID cases and we applied it to three Greek infants with suspected SCID. *Results*: Each patient displayed a distinct immunophenotype—T^−^B^−^NK^−^, T^−^B^−^NK^+^ and T^−^B^+^NK^−^, respectively—and was found to harbor pathogenic or likely pathogenic variants in the analyzed SCID-related genes. In particular, patient 1 carried two heterozygous *ADA* variants (c.58G>A, p.Gly20Arg and c.956_960del, p.Glu319Glyfs); patient 2 harbored two discrete pathogenic variants in the *DCLRE1C* gene (a large deletion of exons 1–3 and the nonsense mutation c.241C>T, p.Arg81*), causing Artemis deficiency; and patient 3 carried a hemizygous *IL2RG* missense variant (c.437T>C, p.Leu146Pro), associated with X-linked SCID. All variants were confirmed by Sanger sequencing. *Conclusions*: Our method successfully identified the underlying genetic defects in all patients, thereby establishing a molecular diagnosis of SCID. These findings highlight the potential of targeted NGS assays for achieving rapid and accurate molecular diagnosis of SCID, which is crucial for the timely treatment of life-threatening conditions in affected children.

## 1. Introduction

Severe combined immunodeficiency (SCID) is one of the most fatal groups of inborn errors of immunity (IEI), characterized by a profound T cell lymphopenia, often accompanied by the absence of B and/or NK cells [1,2]. A considerable number of genes have been implicated in the pathogenesis of SCID. The most prevalent type of SCID worldwide, accounting for approximately half of cases, is X-linked SCID, caused by mutations in the *IL2RG* gene that codes for the common gamma chain protein of the IL-2 receptor. The next most common types of SCID are associated with genetic defects in the *ADA*, *RAG1* and *RAG2* genes. Last but not least, deficiencies in the *DCLRE1C* (Artemis) or more rarely in the *CD3*, *ZAP70* and *IL7RG* genes are also causative of the disease [3].

Early diagnosis of SCID, ideally within the first 3.5 months of life, can be life-saving, as more than 50% of children with SCID may die before a definite diagnosis and therapeutic intervention [4,5,6]. Delayed SCID diagnosis is associated with a poor prognosis along with high morbidity and mortality rates [7,8]. Although ADA-SCID was the first inherited disorder successfully treated with gene therapy [9], the hematopoietic stem cell transplantation (HSCT) remains the only potentially curative treatment for SCID to date, regardless of the underlying molecular defect. Thus, rapid diagnosis of SCID is of utmost importance in terms of disease treatment and outcome.

Advances in molecular diagnostics with the implication of next-generation sequencing (NGS) technologies have allowed the analysis of a large number of candidate genes, thereby facilitating the identification of monogenic defects underlying IEI. As a result, the NGS assay has become a reliable and cost-effective tool for IEI diagnosis [10,11,12,13]. On the other hand, Sanger sequencing of multiple candidate genes as an initial diagnostic approach is rather laborious, time-consuming and expensive, and nowadays, its use has been primarily restricted to confirm NGS results and to investigate the inheritance pattern of the identified mutations in segregation analyses [14].

The purpose of our study was to develop an NGS platform covering the 30 most common genes related to SCID pathogenesis. We achieved an early molecular diagnosis in three infants with SCID clinical and immunological phenotype, suggesting that our NGS assay serves as a rapid and reliable method for SCID diagnosis, allowing for the timely and appropriate management of affected patients.

## 2. Materials and Methods

### 2.1. Patients

Three patients of Greek origin (P1, P2 and P3) with suspected SCID were included in the study.

P1, a 37-day-old male with failure to thrive, exhibited severe leucopenia, neutropenia and lymphopenia. Immunophenotyping revealed complete absence of T and B cells, as well as a profound reduction in NK cells, consistent with a Τ^−^Β^−^ΝΚ^−^ SCID. Additionally, the thymic shadow on chest X-ray was absent.

P2, a 3.5-month-old female, displayed a medical history of fever and lymphopenia at the 50th day of life, which was initially attributed to a viral infection. On the 57th day of life, the fever recurred, and further laboratory analysis demonstrated a cytomegalovirus (CMV) infection of the central nervous system (CNS). After 14 days of intravenous ganciclovir and oral valganciclovir treatment, the patient was discharged from the hospital. However, one month later, fever and lymphopenia recurred and CMV DNA was detected again in both the cerebrospinal fluid (CSF) and the urine. Immunophenotyping of peripheral white blood cells showed absence of T and B cells with normal NK cell counts, consistent with Τ^−^Β^−^ΝΚ^+^ SCID.

P3, an 8.5-month-old male with failure to thrive from the 5th month of life, suffered from a persistent, progressively worsening infection of the lower respiratory tract due to human metapneumovirus (hMPV) for over 2 months. Laboratory tests revealed severe lymphopenia, hypogammaglobulinemia, decreased counts of NK and CD3^+^, CD4^+^ and CD8^+^ T cells, a profound reduction in the number of naïve CD8^+^CD45RA^+^ cytotoxic T cells (2.9%) and naïve CD4^+^CD45RA^+^ T helper cells (0.3%), as well as a remarkable increase in γδ^+^ T cells (mainly memory Vδ1^+^). All the aforementioned findings indicate a Τ^−^Β^+^ΝΚ^−^ SCID phenotype.

Demographic, laboratory and clinical characteristics of all patients are summarized in Table 1. The study was approved by the Ethics Committee of the Medical School of the University of Thessaly (304/11 August 2022) and parents of the patients provided signed inform consent for the genetic analysis.

### 2.2. Molecular Studies

Peripheral blood from each patient equal to 300–500 µL was collected in a BD Mi-crotainer^®^ MAP K2 EDTA tube. Genomic DNA was isolated using NucleoSpin^®^ Tissue kit (Macherey–Nagel GmbH & Co. KG, Düren, Germany) and subsequently, eluted DNA concentration was measured utilizing both the Nanodrop^TM^ Spectrometer and the Qubit4^TM^ Fluorometer with dsDNA HS Assay Kit^TM^ (Thermo Fisher Scientific, Waltham, MA, USA).

Furthermore, we designed an NGS custom panel (CPHS-46379Z-983 QIASeq^®^ Targeted DNA Pro Custom Panel, Qiagen, Hilden, Germany) targeting the coding regions of 30 common genes related to severe combined immunodeficiencies (SCID) or combined immunodeficiencies (CID), as presented in detail in Table 2.

DNA libraries were constructed using the customized QIAseq^®^ Targeted DNA Pro panel Kit, following the protocol proposed by the manufacturer in QIAseq^®^ Targeted DNA Pro Handbook (December 2022, Qiagen). The input DNA amount for all samples was determined at 80 ng. In brief, during library preparation, DNA samples were submitted to enzymatic fragmentation, end-repair and A-tail addition, followed by ligation of the generated fragments with a barcoded-specific adapter, containing the UMIs and sample-specific indices. After a targeted enrichment and a universal PCR, libraries were quantified by qPCR and then equimolarly pooled, in order to achieve an equal representation of all the samples. The pooled libraries were subsequently diluted and finally loaded onto a micro flow cell on a MiSeq Instrument (Illumina, San Diego, CA, USA) to undergo paired-end (2 × 150) sequencing.

Sequencing raw data were aligned to the *Homo sapiens* (human) reference genome GRCh38 (hg38), a process followed by variant calling through smCounter2. For this purpose, QIAGEN GeneGlobe Data Analysis software (https://geneglobe.qiagen.com/, accessed on 6 December 2024) was utilized. Data relating to the pathogenicity of the identified variants were derived from international databases, such as Clinvar (https://www.ncbi.nlm.nih.gov/clinvar/, accessed on 30 March 2025) and Franklin by Genoox (https://franklin.genoox.com, accessed on 30 March 2025), whereas information about minor allele frequencies (MAF) was retrieved from the Genome Aggregation Database (gnomAD, http://gnomad.broadinstitute.org/, accessed on 30 March 2025). In silico evaluation of variant pathogenicity was carried out using SIFT (https://sift.bii.a-star.edu.sg/, accessed on 30 March 2025) and PolyPhen-2 (http://genetics.bwh.harvard.edu/pph2/, accessed on 30 March 2025) prediction tools. Variant classification into pathogenic, likely pathogenic, variant of uncertain significance (VUS), likely benign or benign adhered to the guidelines of the American College of Medical Genetics (ACMG) [16]. Genetic variants were considered as possibly causative of a SCID phenotype when all prediction tools evaluated them as damaging or possibly damaging and/or there was robust literature evidence supporting their pathogenicity. Wild-type and mutated protein 3D structure prediction was performed using AlphaFold 3 software [17], whereas visualization of the detected variants was achieved with PyMOL Molecular Graphics System, Version 3.1.5.1., Schrödinger, LLC, New York, NY, USA.

All variants classified or predicted as pathogenic or likely pathogenic were confirmed by Sanger sequencing. After amplification of the targeted genomic regions with appropriate primers by PCR conducted on a Veriti Thermal Cycler (Applied Biosystems Inc., Foster City, CA, USA), PCR products were purified using a PCR purification kit (Qiagen, Crawley, UK). Purified PCR products were subsequently sequenced using an ABI Prism 310 genetic analyzer (Applied Biosystems Inc., Foster City, CA, USA) and a BigDye Terminator DNA sequencing kit (Applied Biosystems Inc., Foster City, CA, USA). The nucleotide sequences of the primers utilized for the detection of the identified variants along with the corresponding PCR conditions are summarized in Table 3.

## 3. Results

### 3.1. Genetic Analysis of Patient P1

Molecular analysis of patient P1 revealed two heterozygous variants in the *ADA* gene (c.58G>A, p.Gly20Arg and c.956_960del, p.Glu319Glyfs). The first one was a very rare heterozygous missense variant in exon 2, with a frequency lower than 0.01% in the non-Finnish European (NFE) population (gnomAD Exome). The second variant was a frameshift mutation, located in exon 10 and characterized by a deletion of five nucleotides, that is reported in the NFE population with a frequency of approximately 0.02% (gnomAD Exome).

Taking into account that NGS assays cannot determine the *in cis* or *in trans* state of the detected variants and the fact that parental DNA was not initially obtained to perform a segregation analysis, we evaluated serum ADA levels, which were typically very low (2 U/L), being consistent with ADA-SCID. Thus, we could imply that the identified variants were *in trans*. In the following weeks, targeted Sanger sequencing of exon 10 of the *ADA* gene in both parents demonstrated that the healthy father carried ADA:c.956_960del in heterozygous state, thus reinforcing our hypothesis that the index patient was compound heterozygous (Table 4).

#### 3.1.1. Pathogenicity Evaluation of the ADA:c.58G>A Variant

**Figure 1:**
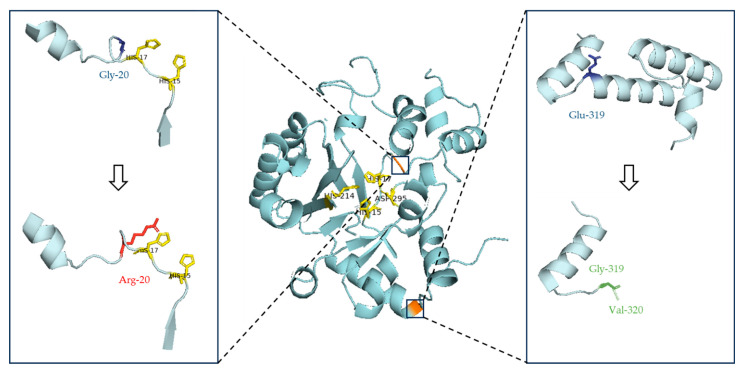
Prediction of the ADA protein structure in patient P1, compared to its wild-type form. ADA enzyme key active sites (His-15, His-17, His-214 and Asp-295 [22]) are highlighted in yellow, wild-type amino acids are depicted in blue, and mutated amino acids in red and green (missense and frameshift mutations, respectively). The orange-colored regions indicate amino acids where changes occur. The missense variant c.58G>A leads to a glycine substitution by arginine at position 20 of the ADA protein, located near the key active sites His-15 and His-17. The second variant detected (c.956_960del) is an open reading frame mutation resulting in a truncated protein with 320 amino acids.

The c.58G>A (p.Gly20Arg) variant is described as pathogenic/likely pathogenic in the ClinVar database (Variation ID: 68267). SIFT and Polyphen-2 predicted the variant as deleterious and probably damaging, respectively. This variant has been already described in homozygosity in two unrelated patients with ADA deficiency, one from a Canada [20] and one from Turkey [21]. A guanine to adenine transition at a possible methylation CpG dinucleotide site results in the substitution of glycine by arginine at amino acid 20 of the ADA enzyme (Figure 1). This missense mutation is detected in a highly conserved area of the ADA protein that is involved in Zn^2+^ binding at the catalytic site, thus impairing ADA enzyme activity [20].

#### 3.1.2. Pathogenicity Evaluation of the ADA:c.956_960del Variant

The c.956_960del defect is classified as pathogenic in ClinVar database (Variation ID: 193544). This frameshift variant produces a premature stop codon at position 319, three codons downstream the mutation site. The result is the creation of an abnormal transcript that may be degraded or may lead to the production of a truncated ADA protein with potentially abnormal function (Figure 1). Loss-of-function variants in the *ADA* gene are known to be pathogenic [23,24]. This pathogenic variant has been previously observed in both homozygous and compound heterozygous patients with ADA-related SCID [23,24,25,26].

### 3.2. Genetic Variations in Patient P2

**Figure 2:**
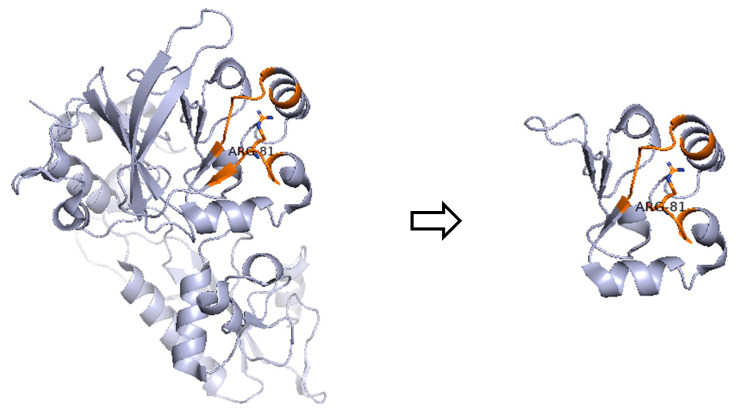
Prediction of the Artemis protein structure in patient P2, compared to its wild-type form. The nonsense variant c.241C>T converts codon CGA that codes for arginine to the stop codon UGA, thereby causing premature termination of the translation process and the production of a truncated protein.

In patient P2, a female infant with persisting CMV infection of the CNS and a Τ^−^Β^−^ΝΚ^+^ SCID phenotype, we detected the nonsense variant c.241C>T (p.Arg81*) in the *DCLRE1C* gene, causing Artemis deficiency. According to ClinVar database (Variation ID: 4665), DCLRE1C:c.241C>T variant meets the criteria to be classified as pathogenic for autosomal recessive SCID due to *DCLRE1C* deficiency. This very rare variant with a frequency of 0.0031% in the non-Finnish European population introduces a premature termination codon in the protein-coding mRNA, hence producing either a truncated (Figure 2) or no protein due to nonsense-mediated decay, which are commonly known mechanisms for disease. Functional studies reveal a damaging effect, including significantly reduced Artemis protein activity [27]. This Artemis-deficiency causing variant has been already reported in the literature in individuals affected with SCID [27,28,29].

Based on the results of our targeted NGS assay, patient P2 was initially identified as homozygous for the previously described variant in the *DCLRE1C* gene. This finding suggested apparent homozygosity, which is consistent with the presence of two identical copies of the same allele. However, genetic analysis of the patient’s parents demonstrated that patient P2 is, in fact, compound heterozygous. More specifically, the maternal allele was found to carry a large deletion encompassing exons 1–3, while the paternal allele harbored the c.241C>T (p.Arg81*) nonsense mutation (Table 4).

### 3.3. Genetic Variations in Patient P3

**Figure 3:**
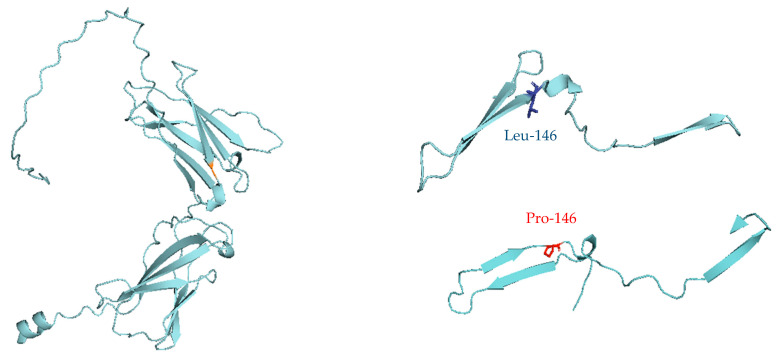
Prediction of the common gamma chain (γc) structure in patient P3, compared to its wild-type form. The missense variant c.437T>C in the *IL2RG* gene causes the substitution of neutral and non-polar leucine (blue) at position 146 of the common gamma chain by neutral and non-polar proline (red).

In patient P3, an 8.5-month-old male infant with persistent, progressively worsening infection of the lower respiratory tract and a Τ^−^Β^+^ΝΚ^−^ SCID phenotype, we identified the c.437T>C (p.Leu146Pro) variant in the *IL2RG* gene. Since *IL2RG* gene is located on the X chromosome, patient P3 was hemizygous for the variant (X-linked SCID). This missense variant causes the substitution of neutral and non-polar leucine at position 146 of the IL2RG protein by proline, which is also a neutral and non-polar amino acid (Figure 3). It is worth mentioning that the detected c.437T>C (p.Leu146Pro) variant represents a *de novo* mutation in the *IL2RG* gene, as no maternal inheritance was confirmed by segregation analysis (Table 4).

Bioinformatic tools predicted the defect as deleterious or probably damaging (SIFT and PolyPhen-2, respectively). Despite the currently available data suggesting that the variant is pathogenic, additional functional studies are necessary to definitively prove that. Hence, this variant has been classified by ClinVar database as likely pathogenic (Variation ID: 2138602). The damaging effects of the IL2RG:c.437T>C variant are probably associated with conformational and/or stability changes in the interleukin-2 receptor gamma chain, which serves as an integral component of multiple cytokine receptors with a pivotal role in the development and function of T and B lymphocytes. At least one publication provides evidence that the aforementioned variant has been already detected in individuals with SCID [30]. No data for this variant is available in population databases.

## 4. Discussion

Our NGS-based diagnostic approach targets 30 genes reported as causative of SCID or CID by the International Union of Immunological Societies [1]. Among these genes, 7 are linked with Τ^−^Β^+^SCID (*IL2RG*, *JAK3*, *IL7R*, *PTPRC*, *CD3D*, *CD3E* and *CD247*), 9 are involved in Τ^−^Β^−^SCID (*RAG1*, *RAG2*, *DCLRE1C*, *PRKDC*, *NHEJ1*, *LIG4*, *ADA*, *AK2* and *RAC2*) and 14 (*ZAP70*, *TAP1*, *TAP2*, *TAPBP*, *B2M*, *CIITA*, *RFXANK*, *RFX5*, *RFXAP*, *DOCK2*, *CD3G*, *MALT1*, *CARD11* and *BCL10*) are associated with combined immunodeficiency, generally less profound than SCID. The panel was thus designed to encompass both classical SCID and less severe CID forms, allowing for a comprehensive molecular diagnosis of patients with suspected cellular and humoral IEI.

A definite molecular diagnosis with a high coverage of targeted genomic regions was achieved for all three patients analyzed. Since the patients’ genotype is consistent with the severely affected clinical phenotype and the laboratory findings observed, their disease could be probably attributed to the genetic variants detected via our targeted NGS-based method. The presence of all variants was further confirmed through a Sanger sequencing validation procedure (Supplementary Figure S1). All these data certify the reliability and validity of our NGS-based protocol in diagnosing SCID.

Segregation analysis revealed the inheritance pattern for three out of four of the detected variants. In more detail, patient P1 inherited the c.956_960del (p.Glu319Glyfs) from his father, while the c.58G>A (p.Gly20Arg) variant may potentially represent a *de novo* mutation or a variant of maternal origin. In the case of patient P2, our NGS-based diagnostic approach initially suggested homozygosity for the detected variant. However, patient P2 was found to be actually compound heterozygous, presenting with two distinct pathogenic variants in the *DCLRE1C* gene: a maternally inherited allele harboring a large deletion of exons 1–3 and a paternally inherited allele carrying the c.241C>T (p.Arg81*) nonsense variant. This finding underlines the limitations of targeted NGS assays in detecting deletions spanning large genomic regions, while confirming biallelic *DCLRE1C* pathogenic variants in patient P2, which is consistent with an autosomal recessive inheritance pattern. Finally, patient P3 seems to be a carrier of a *de novo* mutation c.437T>C (p.Leu146Pro) in the *IL2RG* gene, as no maternal inheritance was observed. In addition, it is well known that approximately one-third of X-linked severe combined immunodeficiency (X-SCID) cases have a positive family history, implying that the rest of the two-thirds arise from *de novo* mutations in the *IL2RG* gene [19].

It is worth mentioning that according to the literature, P1 seems to be the third case of ADA deficiency and P2 the fourth case of Artemis deficiency reported in the Greek population. Finally, P3 likely represents the first documented case of X-linked SCID (IL2RG deficiency) in a Greek infant with maternal Greek origin [31]. This is the first report in Greece, contributing valuable data to the limited genetic SCID/CID registry in the region and supporting the need for a broader application of NGS-based methods in SCID/CID diagnostics. This approach could significantly improve SCID and CID diagnosis, offering a promising path to better outcomes for pediatric patients suffering from such life-threatening disorders.

## 5. Conclusions

The NGS-based diagnostic approach presented herein was capable of identifying four different genetic variants that are probably associated with three distinct monogenic disorders (Artemis, common gamma chain and ADA deficiency). While the rarity of the condition constrained sample availability, leading to a small cohort which included only three patients, our observations provide valuable preliminary insights and lay the groundwork for future validation in larger cohorts. According to these preliminary findings, our molecular approach may be used as a reliable diagnostic tool in patients with suspected SCID/CID. However, further segregation analyses for all variants and functional studies along with the selection of a larger number of SCID/CID-causative candidate genes that could be included in our targeted NGS panel are expected to significantly increase the diagnostic potential of our method.

## Figures and Tables

**Table 1:** Clinical and immunological features of patients subjected to molecular analysis.

Patient	Sex	Age on Diagnosis	Clinical Manifestations	Immune Phenotype	Outcome
P1	M	37 days	Failure to thrive, severe leucopenia, neutropenia and lymphopenia, absence of thymic shadow	Τ^−^Β^−^ΝΚ^−^ SCID	Alive, under enzyme replacement therapy and chemoprotective regimen until gene therapy
P2	F	3.5 months	Persistent CMV infection of the CNS, hypogammaglobulinemia, severe lymphopenia from 50th day of life, normal levels of NK cells	Τ^−^Β^−^ΝΚ^+^ SCID	Death due to HSCT complications and CMV treatment toxicities
P3	M	8.5 months	Persistent respiratory infection, leucopenia, severe lymphopenia, hypogammaglobulinemia, reduced CD3^+^, CD4^+^, CD8^+^ T and NK cells	Τ^−^Β^+^ΝΚ^−^ SCID	Death prior to HSCT due to respiratory failure

Abbreviations: M, male; F, female; *ADA*, adenosine deaminase; *DCLRE1C*, DNA cross-link repair 1C; *IL2RG*, interleukin 2 receptor subunit gamma; CMV, cytomegalovirus; CNS, central nervous system; SCID, severe combined immunodeficiency; HSCT, hematopoietic stem cell transplantation.

**Table 2:** List of the 30 SCID/CID-causative genes included in the NGS targeted panel of the study.

No	Implicated Gene/OMIM	Associated IEI	Clinical Manifestations	Inheritance
1	*IL2RG*/308380	γc deficiency	CID moderate, SCID (T^−^B^+^NK^−^)	XLR
2	*JAK3*/600173	JAK3 deficiency	SCID (T^−^B^+^NK^−^)	AR
3	*IL7R*/146661	IL7Rα deficiency	IMD 104, SCID (T^−^B^+^NK^+^)	AR
4	*PTPRC*/151460	CD45 deficiency	IMD 105, SCID (T^−^B^+^NK^+^)	AR
5	*CD3D*/186790	CD3δ deficiency	IMD19, SCID (T^−^B^+^NK^+^)	AR
6	*CD3E*/186830	CD3ε deficiency	IMD18, SCID (T^−^B^+^NK^+^)	AR
7	*CD247*/186780	CD3ζ deficiency	IMD25 (provisional, T^−^B^+^NK^+^)	AR
8	*RAG1*/179615	RAG deficiency	Combined cellular and humoral immune defects with granulomas, Omenn syndrome, SCID (T^−^B^−^NK^+^)	AR
9	*RAG2*/179616			
10	*DCLRE1C*/605988	Artemis deficiency	Ommen syndrome, Athabascan-type SCID (T^−^B^−^NK^+^)	AR
11	*PRKDC*/600899	DNA-PKcs deficiency	IMD26, with or without neurologic abnormalities, SCID (T^−^B^−^NK^+^)	AR
12	*LIG4*/601837	DNA ligase IV deficiency	LIG4 syndrome, SCID (T^−^B^−^NK^+^)	AR
13	*NHEJ1*/611290	Cernunnos/XLF deficiency	IMD124, SCID (T^−^B^−^NK^+^), Microphthalmia/coloboma 13	AR
14	*AK2*/103020	AK2 defect	Reticular dysgenesis (T^−^B^−^NK^−^)	AR
15	*ADA*/608958	ADA deficiency	SCID due to ADA deficiency (T^−^B^−^NK^−^)	AR
16	*RAC2*/602049	Activated RAC2 defect	IMD73A with defective neutrophil chemotaxis and leukocytosis, IMD73B with defective neutrophil chemotaxis and lymphopenia, SCID (T^−^B^−^NK^−^)	AD GoF
17	*ZAP70*/176947	ZAP-70 deficiency	Autoimmune disease, multisystem, infantile-onset, 2, IMD48	AR
18	*RFX5*/601863	MHC class II deficiency	MHC class II deficiency 3, MHC class II deficiency 5 (provisional)	AR
19	*RFXAP*/601861		MHC class II deficiency 4	
20	*CIITA*/600005		MHC class II deficiency 1	
21	*RFXANK*/603200		MHC class II deficiency 2	
22	*TAP1*/170260	MHC class I deficiency	MHC class I deficiency 1	AR
23	*TAP2*/170261		MHC class I deficiency 2	
24	*TAPBP*/601962		MHC class I deficiency 3 (provisional)	
25	*B2M*/109700		IMD43	
26	*CD3G*/186740	CD3γ deficiency	IMD 17, CD3 gamma deficient	AR
27	*DOCK2*/603122	DOCK2 deficiency	IMD40	AR
28	*CARD11*/607210	CARD11 deficiency	IMD11A	AR LoF
29	*BCL10*/616098	BCL10 deficiency	IMD37 (provisional)	AR
30	*MALT1*/604860	MALT1 deficiency	IMD12	AR

Abbreviations: OMIM, Online Mendelian Inheritance in Man; *IL2RG*, interleukin 2 receptor subunit gamma; *JAK3*, Janus kinase 3; *IL7R*, interleukin-7 receptor; *PTPRC*, protein tyrosine phosphatase receptor type C; *CD3D*, CD3 delta subunit of T-cell receptor complex; *CD3E*, CD3 epsilon subunit of T-cell receptor complex; *CD247*, T-cell surface glycoprotein CD3 zeta chain; *RAG1*, recombination activating gene 1; *RAG2*, recombination activating gene 2; *DCLRE1C*, DNA cross-link repair 1C; *PRKDC*, protein kinase, DNA-activated, catalytic subunit; *LIG4*, DNA ligase 4; *NHEJ1*, non-homologous end joining factor 1; *AK2*, adenylate kinase 2; *ADA*, adenosine deaminase; *RAC2*, rac family small GTPase 2; *ZAP70*, zeta chain of T cell receptor-associated protein kinase 70; *RFX5*, regulatory factor X5; *RFXAP*, regulatory factor X-associated protein; *CIITA*, class II major histocompatibility complex transactivator; *RFXANK*, regulatory factor X-associated ankyrin-containing protein; *TAP1*, transporter 1, ATP binding cassette subfamily B member; *TAP2*, transporter 2, ATP binding cassette subfamily B member; *TAPBP*, TAP binding protein; *B2M*, beta-2-microglobulin; *CD3G*, CD3 gamma subunit of T-cell receptor complex; *DOCK2*, dedicator of cytokinesis 2; *CARD11*, caspase recruitment domain family member 11; *BCL10*, B-cell lymphoma/leukemia 10 immune signaling adaptor; *MALT1*, mucosa-associated lymphoid tissue lymphoma translocation 1, IEI, inborn error of immunity; IL7Rα, interleukin-7 receptor subunit alpha; DNA-PKcs, DNA-dependent protein kinase catalytic subunit; XLF deficiency, XRCC4-like factor deficiency; MHC, major histocompatibility complex; CID, combined immunodeficiency; SCID, severe combined immunodeficiency; XLR, X-linked recessive; AR, autosomal recessive; AD, autosomal dominant; IMD, immunodeficiency; GoF, gain-of-function; LoF, loss-of-function [1,15].

**Table 3:** PCR protocols used for the confirmation of the detected variants in *ADA*, *DCLRE1C* and *IL2RG* genes by Sanger sequencing.

Gene	Targeted Region	Transcript	Primer Sequence	PCR Product (bp)	PCR Conditions	Reference
*ADA*	Exon 2	NM_000022.4	F: 5′-AACATTAAGCTCTGAAAGGTCCTTCG-3′ R: 5′-GCTTGATTCCCACAGGGAGAC-3′	272	94 °C 5 min, 35 cycles (94 °C 30 s, 62 °C 45 s, 72 °C 45 s), 72 °C 10 min	[18]
	Exon 10		F: 5′-GGCTGCCATTCTGCCTGGTT-3′ R: 5′-CCTCTCTCCAAAGATTCCAGGC-3′	495	94 °C 5 min, 40 cycles (94 °C 30 s, 60 °C 45 s, 72 °C 45 s), 72 °C 10 min	
*DCLRE1C*	Exon 3	NM_001033855.3	F: 5′-TCTAACAGATTTTGTGCCAGCG-3′ R: 5′-CTGAAGTATGTTACAAACTGAGGC-3′	413	94 °C 5 min, 32 cycles (94 °C 30 s, 62 °C 1 min, 72 °C 45 s), 72 °C 3 min	In house design of primers
*IL2RG*	Exons 3–4	NM_000206.3	F: 5′-TGCAGTACCCAGATTGGCC-3′ R: 5′-GGCCTTAGCTGCTACATTCACG-3′	625	94 °C 5 min, 36 cycles (94 °C 45 s, 60 °C 1 min, 72 °C 30 s), 72 °C 2 min	[19]

Abbreviations: *ADA*, adenosine deaminase; *DCLRE1C*, DNA cross-link repair 1C; *IL2RG*, interleukin 2 receptor subunit gamma; F, forward; R, reverse; PCR, polymerase chain reaction; bp, base pairs.

**Table 4:** Inheritance pattern of the pathogenic variants detected in the patients subjected to genetic analysis.

Patient	Sex	Immune Phenotype	Gene	Pathogenic Variants	Zygosity	Mode of Inheritance
P1	M	Τ^−^Β^−^ΝΚ^−^ SCID	*ADA*	c.58G>A (p.Gly20Arg)	Het	Not performed (Maternal inheritance or de novo)
				c.956_960del (p.Glu319Glyfs)	Het	Paternal inheritance
P2	F	Τ^−^Β^−^ΝΚ^+^ SCID	*DCLRE1C*	c.241C>T (p.Arg81 *)	Het	Paternal inheritance
				Deletion of exons 1–3	Het	Maternal inheritance
P3	M	Τ^−^Β^+^ΝΚ^−^ SCID	*IL2RG*	c.437T>C (p.Leu146Pro)	Hmz	De novo (Mother did not harbor the pathogenic variant)

Abbreviations: M, male; F, female; SCID, severe combined immunodeficiency; *ADA*, adenosine deaminase; *DCLRE1C*, DNA cross-link repair 1C; *IL2RG*, interleukin 2 receptor subunit gamma; Het, heterozygous; Hmz, hemizygous; * indicates a translation premature termination (stop) codon.

## Data Availability

All data being analyzed in this manuscript are available upon request to the corresponding author.

## Supplementary Materials

The following supporting information can be downloaded at: https://www.mdpi.com/article/10.3390/medicina61091644/s1, Figure S1: Sanger sequencing electropherograms confirming the presence of the variants ADA:c.58G>A, p.Gly20Arg and ADA:c.956_960del, p.Glu319Glyfs (A), DCLRE1C:c.241C>T, p.Arg81* (B) and IL2RG:c.437T>C, p.Leu146Pro (C) in patients P1, P2 and P3, respectively.

## References

[1] Poli M.C., Aksentijevich I., Bousfiha A.A., Cunningham-Rundles C., Hambleton S., Klein C., Morio T., Picard C., Puel A., Rezaei N. (2025). Human inborn errors of immunity: 2024 update on the classification from the International Union of Immunological Societies Expert Committee. J. Hum. Immun..

[2] Barreiros L.A., Sousa J.L., Geier C., Leiss-Piller A., Kanegae M.P.P., França T.T., Boisson B., Lima A.M., Costa-Carvalho B.T., Aranda C.S. (2022). SCID and Other Inborn Errors of Immunity with Low TRECs—The Brazilian Experience. J. Clin. Immunol..

[3] Mikkers H., Pike-Overzet K., Staal F.J.T. (2012). Induced pluripotent stem cells and severe combined immunodeficiency: Merely disease modeling or potentially a novel cure?. Pediatr. Res..

[4] Puck J.M. (2007). Population-based newborn screening for severe combined immunodeficiency: Steps toward implementation. J. Allergy Clin. Immunol..

[5] Brown L., Xu-Bayford J., Allwood Z., Slatter M., Cant A., Davies E.G., Veys P., Gennery A.R., Gaspar H.B. (2011). Neonatal diagnosis of severe combined immunodeficiency leads to significantly improved survival outcome: The case for newborn screening. Blood.

[6] Khalturina E.O., Degtyareva N.D., Bairashevskaia A.V., Mulenkova A.V., Degtyareva A.V. (2021). Modern diagnostic capabilities of neonatal screening for primary immunodeficiencies in newborns. Clin. Exp. Pediatr..

[7] Chan A., Scalchunes C., Boyle M., Puck J.M. (2011). Early vs. delayed diagnosis of severe combined immunodeficiency: A family perspective survey. Clin. Immunol..

[8] Gennery A.R., Slatter M.A., Grandin L., Taupin P., Cant A.J., Veys P., Amrolia P.J., Gaspar H.B., Davies E.G., Friedrich W. (2010). Transplantation of hematopoietic stem cells and long-term survival for primary immunodeficiencies in Europe: Entering a new century, do we do better?. J. Allergy Clin. Immunol..

[9] Kuo C.Y., Kohn D.B. (2016). Gene Therapy for the Treatment of Primary Immune Deficiencies. Curr. Allergy Asthma Rep..

[10] Nijman I.J., van Montfrans J.M., Hoogstraat M., Boes M.L., van de Corput L., Renner E.D., van Zon P., van Lieshout S., Elferink M.G., van der Burg M. (2014). Targeted next-generation sequencing: A novel diagnostic tool for primary immunodeficiencies. J. Allergy Clin. Immunol..

[11] Stoddard J.L., Niemela J.E., Fleisher T.A., Rosenzweig S.D. (2014). Targeted NGS: A Cost-Effective Approach to Molecular Diagnosis of PIDs. Front. Immunol..

[12] Moens L.N., Falk-Sörqvist E., Asplund A.C., Bernatowska E., Smith C.I.E., Nilsson M. (2014). Diagnostics of Primary Immunodeficiency Diseases: A Sequencing Capture Approach. PLoS ONE.

[13] Al-Mousa H., Abouelhoda M., Monies D.M., Al-Tassan N., Al-Ghonaium A., Al-Saud B., Al-Dhekri H., Arnaout R., Al-Muhsen S., Ades N. (2016). Unbiased targeted next-generation sequencing molecular approach for primary immunodeficiency diseases. J. Allergy Clin. Immunol..

[14] Beck T.F., Mullikin J.C. (2016). the NISC Comparative Sequencing Program. Systematic Evaluation of Sanger Validation of Next-Generation Sequencing Variants. Clin. Chem..

[15] About OMIM—OMIM—(OMIM.ORG). https://omim.org/about.

[16] Richards S., Aziz N., Bale S., Bick D., Das S., Gastier-Foster J., Grody W.W., Hegde M., Lyon E., Spector E. (2015). Standards and guidelines for the interpretation of sequence variants: A joint consensus recommendation of the American College of Medical Genetics and Genomics and the Association for Molecular Pathology. Genet. Med..

[17] Abramson J., Adler J., Dunger J., Evans R., Green T., Pritzel A., Ronneberger O., Willmore L., Ballard A.J., Bambrick J. (2024). Accurate structure prediction of biomolecular interactions with AlphaFold 3. Nature.

[18] Hogner S., Lundman E., Strand J., Ytre-Arne M.E., Tangeraas T., Stray-Pedersen A. (2023). Newborn Genetic Screening—Still a Role for Sanger Sequencing in the Era of NGS. Int. J. Neonatal Screen..

[19] Puck J.M., Pepper A.E., Henthorn P.S., Candotti F., Isakov J., Whitwam T., Conley M.E., Fischer R.E., Rosenblatt H.M., Small T.N. (1997). Mutation Analysis of IL2RG in Human X-Linked Severe Combined Immunodeficiency. Blood.

[20] Yang D.R., Huie M.L., Hirschhorn R. (1994). Homozygosity for a missense mutation (G20R) associated with neonatal onset adenosine deaminase-deficient severe combined immunodeficiency (ADA-SCID). Clin. Immunol. Immunopathol..

[21] Firtina S., Ng Y.Y., Ng O.H., Kiykim A., Aydiner E., Nepesov S., Camcioglu Y., Sayar E.H., Reisli I., Torun S.H. (2020). Mutational landscape of severe combined immunodeficiency patients from Turkey. Int. J. Immunogenet..

[22] Rawat R.S., Kumar S. (2023). Understanding the mode of inhibition and molecular interaction of taxifolin with human adenosine deaminase. J. Biomol. Struct. Dyn..

[23] Adams S.P., Wilson M., Harb E., Fairbanks L., Xu-Bayford J., Brown L., Kearney L., Madkaikar M., Gaspar H.B. (2015). Spectrum of mutations in a cohort of UK patients with ADA deficient SCID: Segregation of genotypes with specific ethnicities. Clin. Immunol..

[24] Baffelli R., Notarangelo L.D., Imberti L., Hershfield M.S., Serana F., Santisteban I., Bolda F., Porta F., Lanfranchi A. (2015). Diagnosis, Treatment and Long-Term Follow Up of Patients with ADA Deficiency: A Single-Center Experience. J. Clin. Immunol..

[25] Geelen J., Pfundt R., Meijer J., Verheijen F.W., van Kuilenburg A.B., Warris A., Marcelis C. (2013). Severe phenotype of severe combined immunodeficiency caused by adenosine deaminase deficiency in a patient with a homozygous mutation due to uniparental disomy. J. Allergy Clin. Immunol..

[26] Cagdas D., Cetinkaya P.G., Karaatmaca B., Esenboga S., Tan C., Yılmaz T., Gümüş E., Barış S., Kuşkonmaz B., Ozgur T.T. (2018). ADA Deficiency: Evaluation of the Clinical and Laboratory Features and the Outcome. J. Clin. Immunol..

[27] Felgentreff K., Lee Y.N., Frugoni F., Du L., van der Burg M., Giliani S., Tezcan I., Reisli I., Mejstrikova E., de Villartay J.-P. (2015). Functional analysis of naturally occurring DCLRE1C mutations and correlation with the clinical phenotype of ARTEMIS deficiency. J. Allergy Clin. Immunol..

[28] Moshous D., Callebaut I., de Chasseval R., Corneo B., Cavazzana-Calvo M., Le Deist F., Tezcan I., Sanal O., Bertrand Y., Philippe N. (2001). Artemis, a novel DNA double-strand break repair/V(D)J recombination protein, is mutated in human severe combined immune deficiency. Cell.

[29] Moshous D., Callebaut I., De Chasseval R., Poinsignon C., Villey I., Fischer A., DE Villartay J. (2003). The V(D)J Recombination/DNA Repair Factor Artemis Belongs to the Metallo-β-Lactamase Family and Constitutes a Critical Developmental Checkpoint of the Lymphoid System. Ann. N. Y. Acad. Sci..

[30] Niemela J.E., Puck J.M., Fischer R.E., Fleisher T.A., Hsu A.P. (2000). Efficient detection of thirty-seven new IL2RG mutations in human X-linked severe combined immunodeficiency. Clin. Immunol..

[31] Michos A., Tzanoudaki M., Villa A., Giliani S., Chrousos G., Kanariou M. (2011). Severe combined immunodeficiency in Greek children over a 20-year period: Rarity of γc-chain deficiency (X-linked) type. J. Clin. Immunol..

