# Supplementary material for: Targeted Next-Generation Sequencing in the Molecular Diagnosis of Severe Combined Immunodeficiency
Source: Medicina (Kaunas). 2025 Sep 11;61(9):1644. doi: 10.3390/medicina61091644 (PMC12471661; doi:10.3390/medicina61091644)

Figure S1. Sanger sequencing electropherograms confirming the presence of the variants ADA:c.58G>A, p.Gly20Arg and ADA:c.956_960del, p.Glu319Glyfs (A), DCLRE1C:c.241C>T, p.Arg81* (B) and IL2RG:c.437T>C, p.Leu146Pro (C) in patients P1, P2 and P3, respectively.

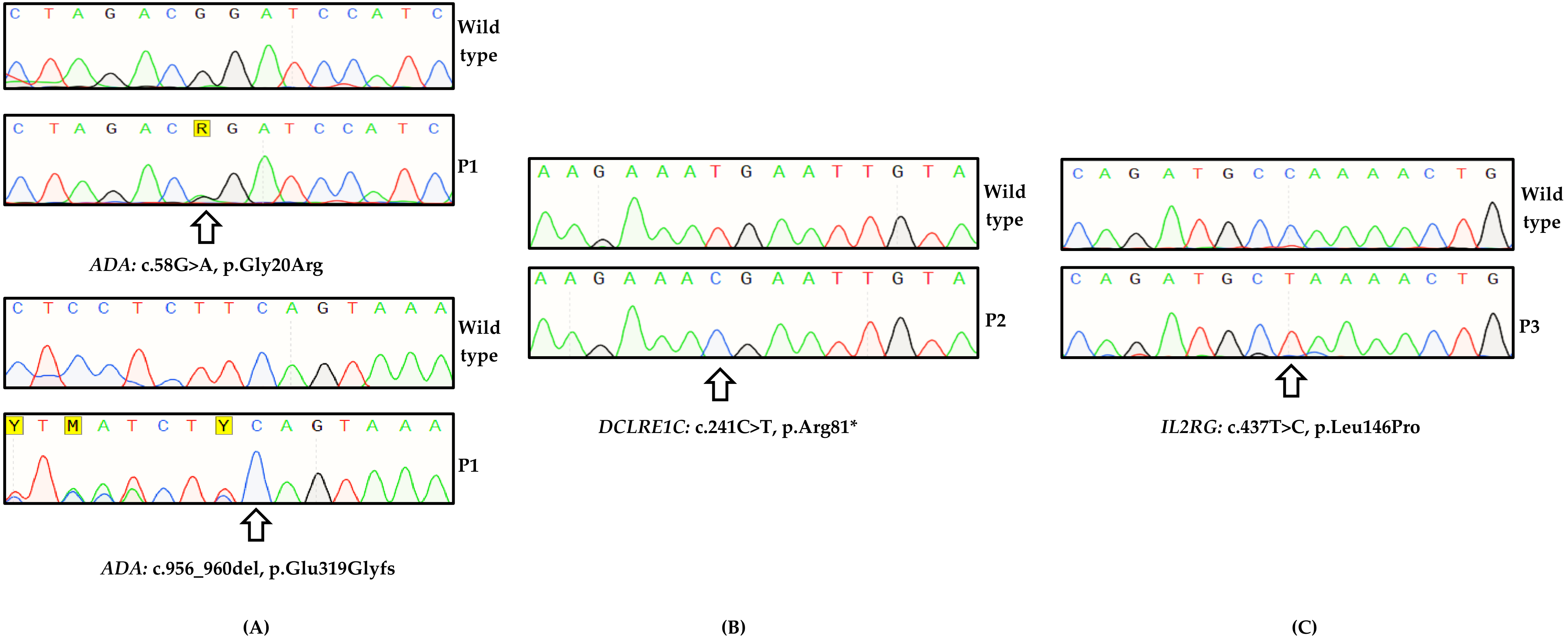

Supplement: Supplementary file 1 [file medicina-61-01644-s001.zip › medicina-3782047-supplementary.pdf]
